# 4-Amino­pyridinium *trans*-diaqua­dioxalatochromate(III) monohydrate

**DOI:** 10.1107/S1600536811044837

**Published:** 2011-11-02

**Authors:** Ichraf Chérif, Jawher Abdelhak, Mohamed Faouzi Zid, Ahmed Driss

**Affiliations:** a Laboratoire de Matériaux et Cristallochimie, Faculté des Sciences de Tunis, Université de Tunis ElManar, 2092 Manar II Tunis, Tunisia

## Abstract

In the non-centrosymmetric structure of the title compound, (C_5_H_7_N_2_)[Cr(C_2_O_4_)_2_(H_2_O)_2_]·H_2_O, the Cr^III^ ion has a slightly distorted octa­hedral coordination environment defined by two chelating oxalato ligands in equatorial positions and two water mol­ecules in axial positions. An extensive three-dimensional network of hydrogen bonds involving all the water mol­ecules, the 4-amino­pyridinium cation and some of the oxalate O atoms contributes to the stabilization of the structure. π–π inter­actions between adjacent pyridine rings provide additional stability of the crystal packing, with a closest distance between pyridine mean planes of 3.613 (1) Å.

## Related literature

For the structural characterization of salts containing the [Cr(C_2_O_4_)_2_(H_2_O)_2_]^−^ anion with various counter-cations, see: Bélombé *et al.* (2009 ▶); Nenwa *et al.* (2010 ▶). For oxalate coord­ination modes, see: Tang *et al.* (2002 ▶); Martak *et al.* (2009 ▶); Hernández-Molina *et al.* (2001 ▶); Zhao *et al.* (2004 ▶). For C—O distances in oxalate anions, see: Marinescu *et al.* (2000 ▶). For geometric parameters of the 4-amino­pyridinium cation, see: Fun *et al.* (2008 ▶, 2009 ▶, 2010 ▶); Jebas *et al.* (2009 ▶); Quah *et al.* (2008 ▶); Ramesh *et al.* (2010 ▶); Rotondo *et al.* (2009 ▶); Pan *et al.* (2008 ▶). For discussion of hydrogen bonding, see: Blessing (1986 ▶); Brown (1976 ▶).
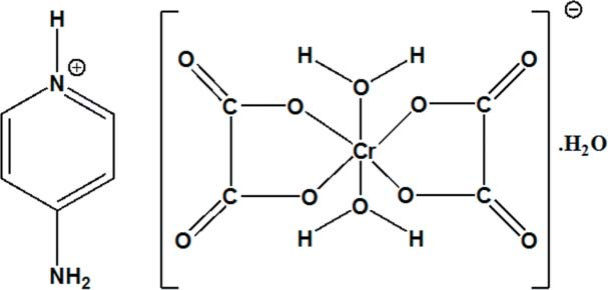


## Experimental

### 

#### Crystal data

(C_5_H_7_N_2_)[Cr(C_2_O_4_)_2_(H_2_O)_2_]·H_2_O
*M* *_r_* = 377.21
Orthorhombic, 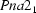

*a* = 21.102 (4) Å
*b* = 9.487 (3) Å
*c* = 7.226 (2) Å
*V* = 1446.7 (7) Å^3^
*Z* = 4
Mo *K*α radiation
μ = 0.85 mm^−1^
*T* = 298 K
0.54 × 0.18 × 0.12 mm

#### Data collection

Enraf–Nonius CAD-4 diffractometer
Absorption correction: ψ scan (North *et al.*, 1968 ▶) *T*_min_ = 0.832, *T*_max_ = 0.903
3729 measured reflections
3150 independent reflections
2857 reflections with *I* > 2σ(*I*)
*R*_int_ = 0.017
2 standard reflections every 120 min  intensity decay: 2.9%

#### Refinement

*R*[*F* ^2^ > 2σ(*F* ^2^)] = 0.025
*wR*(*F* ^2^) = 0.066
*S* = 1.04
3150 reflections
261 parameters
1 restraint
All H-atom parameters refined
Δρ_max_ = 0.30 e Å^−3^
Δρ_min_ = −0.31 e Å^−3^
Absolute structure: Flack (1983 ▶), 1447 Friedel pairs
Flack parameter: 0.000 (16)

### 

Data collection: *CAD-4 EXPRESS* (Duisenberg, 1992 ▶; Macíček & Yordanov, 1992 ▶); cell refinement: *CAD-4 EXPRESS*; data reduction: *XCAD4* (Harms & Wocadlo, 1995 ▶); program(s) used to solve structure: *SHELXS97* (Sheldrick, 2008 ▶); program(s) used to refine structure: *SHELXL97* (Sheldrick, 2008 ▶); molecular graphics: *DIAMOND* (Brandenburg, 1998 ▶); software used to prepare material for publication: *WinGX* (Farrugia, 1999 ▶).

## Supplementary Material

Crystal structure: contains datablock(s) I, global. DOI: 10.1107/S1600536811044837/wm2545sup1.cif

Structure factors: contains datablock(s) I. DOI: 10.1107/S1600536811044837/wm2545Isup2.hkl

Additional supplementary materials:  crystallographic information; 3D view; checkCIF report

## Figures and Tables

**Table 1:** Selected bond lengths (Å)

Cr1—O1	1.9636 (15)
Cr1—O4	1.9658 (15)
Cr1—O2	1.9715 (16)
Cr1—O3	1.9810 (16)
Cr1—O*W*1	1.9847 (17)
Cr1—O*W*2	2.0079 (18)

**Table 2:** Hydrogen-bond geometry (Å, °)

*D*—H⋯*A*	*D*—H	H⋯*A*	*D*⋯*A*	*D*—H⋯*A*
O*W*3—H*W*31⋯O8^i^	0.78 (4)	2.06 (4)	2.823 (3)	167 (4)
O*W*3—H*W*32⋯O8^ii^	0.93 (4)	1.93 (4)	2.843 (3)	169 (4)
O*W*2—H*W*21⋯O7^iii^	0.89 (4)	1.78 (4)	2.640 (3)	161 (3)
O*W*2—H*W*22⋯O9^iv^	0.74 (4)	2.07 (4)	2.784 (3)	162 (4)
O*W*1—H*W*11⋯O*W*3^v^	0.90 (4)	1.67 (4)	2.572 (3)	172 (4)
O*W*1—H*W*12⋯O10^vi^	0.76 (5)	2.01 (5)	2.745 (3)	162 (5)
N2—H2⋯O10^vii^	0.93 (4)	2.01 (4)	2.940 (4)	179 (4)
N1—H1⋯O3	1.00 (4)	2.27 (4)	3.061 (3)	135 (4)
N1—H1⋯O9^vi^	1.00 (4)	2.22 (4)	2.974 (3)	131 (4)

Symmetry codes: (i) 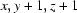; (ii) 
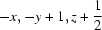
; (iii) 
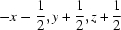
; (iv) 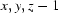; (v) 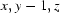; (vi) 
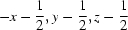
; (vii) 
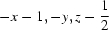
.

**Fig. 1.**
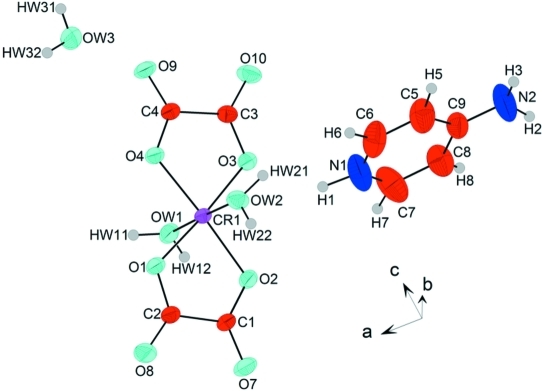
: The asymmetric unit of (C5H7N2)[Cr(C2O4)2(H2O)2].H2O. Thermal displacement parameters are drawn at the 50% probability level; H atoms are given as spheres of arbitrary radius.

**Fig. 2.**
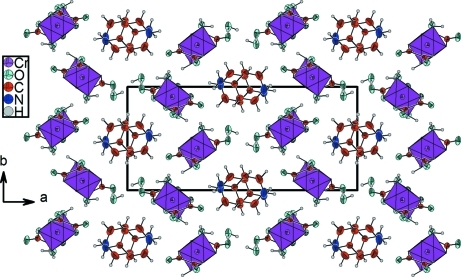
: Projection of the (C5H7N2)[Cr(C2O4)2(H2O)2].H2O structure along the c axis.

**Fig. 3.**
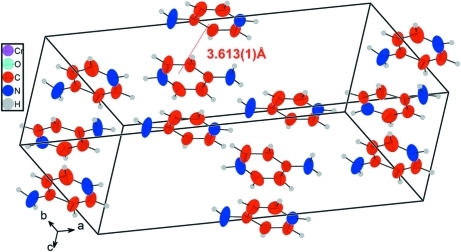
: Perspective view showing the π—π interactions between adjacent 4-aminopyridinium groups. [Cr(C2O4)2(H2O)2]- anions and uncoordinated water molecules have been omitted for clarity.

## supplementary crystallographic information

### Comment

It is well established that the strategy of utilizing transition metals with versatile multidentate ligands like oxalate anions affords various structural topologies (Tang *et al.*, 2002; Martak *et al.*, 2009; Hernández-Molina *et al.*, 2001; Zhao *et al.*, 2004). In this article, we describe the crystal structure of a new chromium(III) salt, (C_5_H_7_N_2_)[Cr(C_2_O_4_)_2_(H_2_O)_2_]^.^H_2_O, containing oxalate (ox) anions which act as chelating ligands.

The structure of the title compound consists of discrete [Cr(C_2_O_4_)_2_(H_2_O)_2_]^-^ mononuclear anions, 4-aminopyridinium cations and uncoordinated water molecules (Fig. 1). In the [Cr(C_2_O_4_)_2_(H_2_O)_2_]^-^ anion, the Cr^III^ atom is six-coordinated in a slightly distorted octahedral coordination environment defined by two chelating bidentate oxalate anions and two water molecules in *trans* position. The expected 90° bond angles vary from 82.13 (6)° to 101.02 (6)°, and the ideal 180° bond angles vary from 175.74 (7)° to 178.68 (8)°. The four Cr—O_(ox)_ bond lengths are different and slightly shorter than the Cr—O_(water)_ bond lengths. This situation was previously observed in homologous salts involving quinolinium (C_9_H_8_N)^+^ and 4-dimethylaminopyridinium (C_7_H_11_N_2_)^+^ counter cations (Bélombé *et al.*, 2009; Nenwa *et al.*, 2010). The C—C bond lengths in the oxalate ligand are as expected for single C—C bonds [1.553 (3) and 1.558 (3) Å for C1—C2 and C3—C4, respectively]. The bond length values of the peripheral and inner C—O bonds compare well with those reported for other oxalate complexes (Marinescu *et al.*, 2000), the shorter values being due to the greater double bond character of the free C—O bonds. The uncoordinated 4-aminopyridinium cations are located between the anions (Fig. 2), their geometric parameters do not show unusual features, they are in accordance with those previously reported (Fun *et al.*, 2008, 2009, 2010; Jebas *et al.*, 2009; Quah *et al.*, 2008; Ramesh *et al.*, 2010; Rotondo *et al.*, 2009; Pan *et al.*, 2008).

The crystal structure is stabilized by strong intermolecular hydrogen bonds (Blessing, 1986; Brown, 1976). (C_5_H_7_N_2_)^+^ and [Cr(C_2_O_4_)_2_(H_2_O)_2_]^-^ are connected *via* N—H···O hydrogen bonds that link the amino N2—H2 group to the free O10 oxalato O atoms. The uncoordinated water molecule functions as both acceptor and donor while the coordinated water molecules function only as donors. Thus, all the constituents of (C_5_H_7_N_2_)[Cr(C_2_O_4_)_2_(H_2_O)_2_]^.^H_2_O are connected to form a three-dimensional structure. Furthermore, π—π interactions between adjacent pyridine rings help to stabilize the crystal packing, the closest distance between two pyridine mean planes being 3.613 (1) Å (Fig. 3).

### Experimental

Aqueous solutions of oxalic acid dihydrate, H_2_C_2_O_4_^.^2H_2_O (2 mmol), and 4-aminopyridine (1 mmol) were added to Cr(NO_3_)_3_^.^9H_2_O (1 mmol) dissolved in 10 mL of water under continuous stirring at 323K. Slow evaporation of the resultant solution led to violet single crystals suitable for X-ray diffraction.

### Refinement

The hydrogen atoms were located in difference Fourier maps. Their displacement parameters were refined isotropically.

### Crystal data

**Table:**

(C_5_H_7_N_2_)[Cr(C_2_O_4_)_2_(H_2_O)_2_]·H_2_O	*F*(000) = 772
*M**_r_* = 377.21	*D*_x_ = 1.732 Mg m^−^^3^
Orthorhombic, *P**n**a*2_1_	Mo *K*α radiation, λ = 0.71073 Å
Hall symbol: P 2c -2n	Cell parameters from 25 reflections
*a* = 21.102 (4) Å	θ = 10–15°
*b* = 9.487 (3) Å	µ = 0.85 mm^−^^1^
*c* = 7.226 (2) Å	*T* = 298 K
*V* = 1446.7 (7) Å^3^	Prism, violet
*Z* = 4	0.54 × 0.18 × 0.12 mm

### Data collection

**Table:**

Enraf–Nonius CAD-4 diffractometer	2857 reflections with *I* > 2σ(*I*)
Radiation source: fine-focus sealed tube	*R*_int_ = 0.017
graphite	θ_max_ = 27.0°, θ_min_ = 2.4°
ω/2θ scans	*h* = −26→1
Absorption correction: ψ scan (North *et al.*, 1968)	*k* = −1→12
*T*_min_ = 0.832, *T*_max_ = 0.903	*l* = −9→9
3729 measured reflections	2 standard reflections every 120 min
3150 independent reflections	intensity decay: 2.9%

### Refinement

**Table:**

Refinement on *F*^2^	Hydrogen site location: difference Fourier map
Least-squares matrix: full	All H-atom parameters refined
*R*[*F*^2^ > 2σ(*F*^2^)] = 0.025	*w* = 1/[σ^2^(*F*_o_^2^) + (0.0364*P*)^2^ + 0.1566*P*] where *P* = (*F*_o_^2^ + 2*F*_c_^2^)/3
*wR*(*F*^2^) = 0.066	(Δ/σ)_max_ < 0.001
*S* = 1.04	Δρ_max_ = 0.30 e Å^−^^3^
3150 reflections	Δρ_min_ = −0.31 e Å^−^^3^
261 parameters	Extinction correction: *SHELXL97* (Sheldrick, 2008), Fc^*^=kFc[1+0.001xFc^2^λ^3^/sin(2θ)]^-1/4^
1 restraint	Extinction coefficient: 0.0022 (7)
Primary atom site location: structure-invariant direct methods	Absolute structure: Flack (1983), 1447 Friedel pairs
Secondary atom site location: difference Fourier map	Flack parameter: 0.000 (16)

### Special details

**Table:**

Geometry. All esds (except the esd in the dihedral angle between two l.s. planes) are estimated using the full covariance matrix. The cell esds are taken into account individually in the estimation of esds in distances, angles and torsion angles; correlations between esds in cell parameters are only used when they are defined by crystal symmetry. An approximate (isotropic) treatment of cell esds is used for estimating esds involving l.s. planes.
Refinement. Refinement of F^2^ against ALL reflections. The weighted R-factor wR and goodness of fit S are based on F^2^, conventional R-factors R are based on F, with F set to zero for negative F^2^. The threshold expression of F^2^ > 2sigma(F^2^) is used only for calculating R-factors(gt) etc. and is not relevant to the choice of reflections for refinement. R-factors based on F^2^ are statistically about twice as large as those based on F, and R- factors based on ALL data will be even larger.

### Fractional atomic coordinates and isotropic or equivalent isotropic displacement parameters (Å^2^)

**Table:**

	*x*	*y*	*z*	*U*_iso_*/*U*_eq_	
Cr1	−0.196274 (13)	0.09444 (3)	−0.00116 (5)	0.01985 (9)	
O9	−0.19814 (7)	0.26620 (15)	0.5002 (3)	0.0295 (3)	
O3	−0.27673 (7)	0.09078 (16)	0.1400 (2)	0.0262 (3)	
O1	−0.11408 (7)	0.10787 (17)	−0.1273 (2)	0.0264 (3)	
OW1	−0.17053 (9)	−0.08746 (18)	0.1118 (2)	0.0283 (3)	
O4	−0.16685 (7)	0.19731 (16)	0.2188 (2)	0.0248 (3)	
OW2	−0.22205 (8)	0.27650 (18)	−0.1212 (2)	0.0295 (3)	
O7	−0.17687 (8)	−0.09782 (17)	−0.4830 (3)	0.0340 (4)	
O2	−0.22078 (7)	−0.00965 (16)	−0.2262 (2)	0.0243 (3)	
O10	−0.31987 (7)	0.17303 (18)	0.4006 (2)	0.0337 (4)	
O8	−0.06261 (8)	0.0179 (2)	−0.3698 (3)	0.0430 (5)	
C3	−0.27494 (10)	0.1550 (2)	0.2972 (3)	0.0228 (4)	
C1	−0.17435 (10)	−0.0316 (2)	−0.3382 (3)	0.0222 (4)	
C4	−0.20797 (10)	0.2123 (2)	0.3479 (3)	0.0218 (4)	
C2	−0.11043 (10)	0.0354 (2)	−0.2779 (3)	0.0252 (4)	
C6	−0.42595 (16)	0.1102 (4)	0.0242 (6)	0.0625 (10)	
C8	−0.51027 (14)	−0.1061 (3)	0.0042 (7)	0.0524 (7)	
N1	−0.40576 (12)	−0.0224 (3)	0.0161 (6)	0.0657 (8)	
N2	−0.59497 (12)	0.0580 (3)	0.0089 (8)	0.0750 (10)	
C9	−0.53327 (11)	0.0317 (3)	0.0117 (5)	0.0413 (5)	
C7	−0.44661 (16)	−0.1296 (4)	0.0020 (8)	0.0726 (10)	
OW3	−0.06036 (9)	0.9143 (3)	0.2640 (3)	0.0449 (5)	
C5	−0.48794 (15)	0.1402 (3)	0.0211 (7)	0.0597 (8)	
H8	−0.5357 (18)	−0.181 (4)	0.003 (6)	0.079 (11)*	
H2	−0.6222 (18)	−0.014 (4)	−0.026 (6)	0.076 (12)*	
H1	−0.359 (2)	−0.038 (5)	0.028 (7)	0.098 (14)*	
HW31	−0.0613 (16)	0.955 (4)	0.358 (6)	0.047 (10)*	
HW21	−0.2581 (18)	0.323 (4)	−0.100 (5)	0.068 (11)*	
HW11	−0.1303 (19)	−0.085 (4)	0.155 (5)	0.058 (10)*	
H6	−0.3942 (19)	0.183 (4)	0.031 (6)	0.079 (12)*	
HW32	−0.022 (2)	0.948 (4)	0.220 (6)	0.077 (12)*	
H5	−0.5032 (19)	0.233 (4)	0.043 (5)	0.077 (12)*	
H3	−0.606 (2)	0.147 (5)	0.010 (7)	0.094 (13)*	
H7	−0.428 (2)	−0.226 (5)	0.001 (8)	0.110 (15)*	
HW12	−0.180 (2)	−0.146 (5)	0.045 (7)	0.094 (16)*	
HW22	−0.2234 (18)	0.269 (4)	−0.223 (6)	0.070 (13)*	

### Atomic displacement parameters (Å^2^)

**Table:**

	*U*^11^	*U*^22^	*U*^33^	*U*^12^	*U*^13^	*U*^23^
Cr1	0.01992 (14)	0.02436 (15)	0.01528 (14)	−0.00098 (11)	0.00098 (14)	−0.00316 (16)
O9	0.0352 (7)	0.0347 (7)	0.0187 (6)	−0.0031 (6)	0.0000 (7)	−0.0086 (10)
O3	0.0232 (7)	0.0314 (8)	0.0241 (8)	−0.0029 (6)	0.0011 (6)	−0.0063 (7)
O1	0.0233 (7)	0.0359 (9)	0.0200 (7)	−0.0051 (6)	0.0011 (6)	−0.0079 (6)
OW1	0.0317 (9)	0.0281 (8)	0.0253 (8)	0.0011 (7)	−0.0057 (7)	−0.0039 (6)
O4	0.0227 (7)	0.0314 (7)	0.0203 (7)	−0.0045 (6)	0.0026 (5)	−0.0064 (6)
OW2	0.0336 (8)	0.0330 (9)	0.0218 (8)	0.0075 (7)	0.0028 (7)	0.0014 (7)
O7	0.0371 (8)	0.0425 (8)	0.0225 (9)	−0.0094 (7)	0.0013 (9)	−0.0108 (8)
O2	0.0229 (7)	0.0299 (8)	0.0202 (6)	−0.0049 (6)	−0.0002 (6)	−0.0026 (6)
O10	0.0303 (8)	0.0364 (9)	0.0344 (9)	−0.0051 (7)	0.0134 (7)	−0.0095 (7)
O8	0.0265 (9)	0.0681 (13)	0.0345 (9)	−0.0075 (9)	0.0089 (7)	−0.0186 (9)
C3	0.0259 (10)	0.0191 (10)	0.0234 (10)	−0.0012 (8)	0.0037 (8)	−0.0008 (8)
C1	0.0266 (10)	0.0228 (9)	0.0172 (9)	−0.0029 (8)	−0.0010 (8)	−0.0013 (8)
C4	0.0267 (10)	0.0201 (9)	0.0184 (9)	0.0004 (8)	−0.0002 (8)	−0.0006 (8)
C2	0.0250 (10)	0.0308 (11)	0.0198 (10)	−0.0021 (9)	0.0015 (8)	−0.0043 (8)
C6	0.0454 (16)	0.075 (2)	0.067 (3)	−0.0191 (16)	−0.0159 (18)	0.013 (2)
C8	0.0456 (14)	0.0354 (13)	0.0761 (19)	0.0017 (11)	−0.001 (2)	0.0092 (18)
N1	0.0334 (12)	0.086 (2)	0.077 (2)	0.0050 (13)	−0.0007 (15)	0.025 (2)
N2	0.0337 (11)	0.0442 (14)	0.147 (3)	0.0059 (11)	−0.019 (2)	−0.022 (2)
C9	0.0334 (11)	0.0362 (12)	0.0542 (15)	0.0001 (9)	−0.0069 (14)	−0.0041 (15)
C7	0.0562 (18)	0.0569 (18)	0.105 (3)	0.0222 (15)	0.009 (3)	0.018 (3)
OW3	0.0304 (10)	0.0753 (15)	0.0290 (9)	0.0014 (10)	−0.0009 (8)	−0.0084 (10)
C5	0.0484 (15)	0.0403 (14)	0.090 (3)	−0.0076 (12)	−0.0156 (19)	−0.002 (2)

### Geometric parameters (Å, °)

**Table:**

Cr1—O1	1.9636 (15)	C3—C4	1.558 (3)
Cr1—O4	1.9658 (15)	C1—C2	1.553 (3)
Cr1—O2	1.9715 (16)	C6—N1	1.330 (5)
Cr1—O3	1.9810 (16)	C6—C5	1.339 (5)
Cr1—OW1	1.9847 (17)	C6—H6	0.96 (4)
Cr1—OW2	2.0079 (18)	C8—C7	1.362 (4)
O9—C4	1.231 (3)	C8—C9	1.396 (4)
O3—C3	1.289 (3)	C8—H8	0.89 (4)
O1—C2	1.290 (3)	N1—C7	1.337 (5)
OW1—HW11	0.91 (4)	N1—H1	1.00 (5)
OW1—HW12	0.77 (5)	N2—C9	1.326 (3)
O4—C4	1.282 (2)	N2—H2	0.92 (4)
OW2—HW21	0.89 (4)	N2—H3	0.88 (4)
OW2—HW22	0.74 (4)	C9—C5	1.407 (4)
O7—C1	1.222 (3)	C7—H7	1.00 (5)
O2—C1	1.287 (3)	OW3—HW31	0.78 (4)
O10—C3	1.219 (3)	OW3—HW32	0.93 (4)
O8—C2	1.219 (3)	C5—H5	0.95 (4)
			
O1—Cr1—O4	93.67 (6)	O2—C1—C2	114.75 (17)
O1—Cr1—O2	83.18 (6)	O9—C4—O4	125.64 (19)
O4—Cr1—O2	176.78 (6)	O9—C4—C3	120.54 (18)
O1—Cr1—O3	175.74 (7)	O4—C4—C3	113.82 (16)
O4—Cr1—O3	82.13 (6)	O8—C2—O1	125.6 (2)
O2—Cr1—O3	101.02 (6)	O8—C2—C1	120.68 (19)
O1—Cr1—OW1	90.32 (7)	O1—C2—C1	113.75 (18)
O4—Cr1—OW1	90.73 (7)	N1—C6—C5	120.9 (3)
O2—Cr1—OW1	88.59 (7)	N1—C6—H6	117 (2)
O3—Cr1—OW1	90.43 (7)	C5—C6—H6	122 (2)
O1—Cr1—OW2	89.02 (7)	C7—C8—C9	119.8 (3)
O4—Cr1—OW2	90.45 (7)	C7—C8—H8	118 (2)
O2—Cr1—OW2	90.20 (8)	C9—C8—H8	123 (2)
O3—Cr1—OW2	90.31 (7)	C6—N1—C7	121.1 (3)
OW1—Cr1—OW2	178.68 (8)	C6—N1—H1	117 (3)
C3—O3—Cr1	114.84 (14)	C7—N1—H1	122 (3)
C2—O1—Cr1	114.20 (13)	C9—N2—H2	118 (2)
Cr1—OW1—HW11	112 (2)	C9—N2—H3	117 (3)
Cr1—OW1—HW12	107 (4)	H2—N2—H3	123 (4)
HW11—OW1—HW12	119 (4)	N2—C9—C8	121.2 (2)
C4—O4—Cr1	115.46 (13)	N2—C9—C5	122.0 (3)
Cr1—OW2—HW21	126 (2)	C8—C9—C5	116.8 (3)
Cr1—OW2—HW22	111 (3)	N1—C7—C8	120.7 (3)
HW21—OW2—HW22	100 (4)	N1—C7—H7	116 (3)
C1—O2—Cr1	113.56 (13)	C8—C7—H7	123 (3)
O10—C3—O3	125.7 (2)	HW31—OW3—HW32	98 (4)
O10—C3—C4	120.81 (18)	C6—C5—C9	120.6 (3)
O3—C3—C4	113.51 (17)	C6—C5—H5	122 (2)
O7—C1—O2	126.0 (2)	C9—C5—H5	117 (2)
O7—C1—C2	119.22 (19)		

### Hydrogen-bond geometry (Å, °)

**Table:**

*D*—H···*A*	*D*—H	H···*A*	*D*···*A*	*D*—H···*A*
OW3—HW31···O8^i^	0.78 (4)	2.06 (4)	2.823 (3)	167 (4)
OW3—HW32···O8^ii^	0.93 (4)	1.93 (4)	2.843 (3)	169 (4)
OW2—HW21···O7^iii^	0.89 (4)	1.78 (4)	2.640 (3)	161 (3)
OW2—HW22···O9^iv^	0.74 (4)	2.07 (4)	2.784 (3)	162 (4)
OW1—HW11···OW3^v^	0.90 (4)	1.67 (4)	2.572 (3)	172 (4)
OW1—HW12···O10^vi^	0.76 (5)	2.01 (5)	2.745 (3)	162 (5)
N2—H2···O10^vii^	0.93 (4)	2.01 (4)	2.940 (4)	179 (4)
N1—H1···O3	1.00 (4)	2.27 (4)	3.061 (3)	135 (4)
N1—H1···O9^vi^	1.00 (4)	2.22 (4)	2.974 (3)	131 (4)

Symmetry codes: (i) *x*, *y*+1, *z*+1; (ii) −*x*, −*y*+1, *z*+1/2; (iii) −*x*−1/2, *y*+1/2, *z*+1/2; (iv) *x*, *y*, *z*−1; (v) *x*, *y*−1, *z*; (vi) −*x*−1/2, *y*−1/2, *z*−1/2; (vii) −*x*−1, −*y*, *z*−1/2.
